# Natural course of Fabry disease with the p. Arg227Ter (p.R227*) mutation in Finland: Fast study

**DOI:** 10.1002/mgg3.930

**Published:** 2019-08-14

**Authors:** Päivi Pietilä‐Effati, Jukka T. Saarinen, Eliisa Löyttyniemi, Reijo Autio, Maria Saarenhovi, Maria K. Haanpää, Ilkka Kantola

**Affiliations:** ^1^ Department of Cardiology Vaasa Central Hospital Vaasa Finland; ^2^ Department of Neurology Vaasa Central Hospital Vaasa Finland; ^3^ Department of Biostatistics University of Turku Turku Finland; ^4^ Department of Radiology Vaasa Central Hospital Vaasa Finland; ^5^ Department of Clinical Physiology and Nuclear Medicine Turku University Hospital, University of Turku Turku Finland; ^6^ Department of Clinical Genetics Turku University Hospital Turku Finland; ^7^ Division of Medicine Turku University Hospital, University of Turku Turku Finland

**Keywords:** cardiac hypertrophy, disease progression, Fabry disease, gender, genotype, late‐onset, phenotype

## Abstract

**Background:**

Fabry disease is caused by a deficient or an absent alfa‐galactosidase A activity and is an X‐linked disorder that results in organ damage and a shortened life span, especially in males. The severity of the disease depends on the type of mutation, gender, skewed X‐chromosome inactivation, and other still unknown factors.

**Methods:**

In this article, we describe the natural course of a common classic Fabry disease mutation, p.Arg227Ter or p.R227*, in Finland.

**Results:**

Four males and ten females belonged to two extended families. The mean age was 46 years (SD 18.4). Six patients (43%) had cardiac hypertrophy, three patients (21%) had ischemic stroke, and none had severe kidney dysfunction. Three patients had atrial fibrillation; two patients who had atrial fibrillation also had pacemakers. All males over 30 years of age had at least one of the following manifestations: cardiac hypertrophy, stroke, or proteinuria. In females, the severity of Fabry disease varied from classic multiorgan disease to a condition that mimicked the attenuated cardiac variant. No one was totally asymptomatic without any signs of Fabry disease. Cardiac magnetic resonance imaging was performed on nine of 14 patients was the most sensitive for detecting early cardiac manifestations. Five patients (55%) had late gadolinium enhancement‐positive segments.

**Conclusion:**

Cardiac involvement should be effectively detected in females before considering them asymptomatic mutation carriers.

## 1 INTRODUCTION

Fabry disease (OMIM # 301500) is a rare X‐chromosome‐linked lysosomal storage disease. A mutation in a gene that encodes enzyme α‐galactosidase A (GLA; OMIM #300644; HGNC 4296; the GenBank reference sequence NM_000169.2) causes impaired catabolism of globotriaosylceramide (Gb3) and an accumulation of Gb3 and its metabolite globotriaosylshingosine (lysoGb3) in several organs (Desnick, Ioannou, & Eng, 2001; Talbot, Nicholls, Fletcher, & Fuller, 2017). Pathological deposits are situated in vascular endothelial cells and in other cell types, such as cardiomyocytes, Schwann cells, dorsal root ganglia, and podocytes, resulting in progressive organ dysfunction and ultimately organ failure (Schiffmann, 2009). However, in the attenuated nonclassical Fabry disease with cardiac predominance, endothelial deposits in myocardial capillaries are absent, and pathological lysosomal inclusions with lamellated cytoplasmic figures are visible only in myocytes (Nakao et al., 1995; von Scheidt et al., 1991). Attenuated variants with renal predominance have also been described (Lavalle et al., 2018; Sugarman, Choudhury, & Jovanovic, 2018). Typical signs and symptoms in the early phase of the classical phenotype are acroparesthesia, episodes of diarrhea and abdominal pain, cornea verticillata, dyshidrosis, tinnitus, and angiokeratomas (Desnick et al., 2001; Eng et al., 2007; Schiffmann, 2009). Severe organ manifestations become evident in the third decade of life in males and approximately the fourth decade of life in females (MacDermot, Holmes, & Miners, 2001a, 2001b; Wilcox et al., 2008). Cardiac hypertrophy, renal dysfunction, and stroke are the most severe complications (Eng et al., 2007). In the attenuated nonclassical disease, mainly heart‐related signs and symptoms are present, with significant cardiac hypertrophy being the most important finding (Nakao et al., 1995; von Scheidt et al., 1991). Life expectancy in male classical Fabry patients is shortened by 15–17 years (MacDermot, Holmes, & Miners, 2001b; Waldek, Patel, Banikazemi, Lemay, & Lee, 2009). In contrast to males, females with classical Fabry disease present a more heterogeneous clinical picture and variable disease progression. The average life spans of classical and nonclassical females and nonclassical males are also somewhat shorter than that of the general population (Arends et al., 2017; MacDermot, Holmes, & Miners, 2001a; Waldek et al., 2009). The variation in genotype–phenotype correlation might be due to X‐chromosome inactivation and variable residual enzymatic activity (Lenders et al., 2016). More recently, Juchniewicz et al. (2018) published a study that notes contradictory findings regarding X‐chromosome inactivation and its correlation with the phenotype.

Almost 1,000 mutations in the GLA gene are recognized today (Stenson et al., 2017). The Fabry follow‐up single mutation study in Finland (FAST) seeks to obtain data to help address the gap in genotype–phenotype correlation in the nonsense mutation R227* (c.679C>T [p.Arg227Ter]), which is the most common mutations causing a classical Fabry disease phenotype in Finland and one of the most common mutations worldwide (Eng, Resnick‐Silverman, Niehaus, Astrin, & Desnick, 1993; Roberto, Michael, & Derralynn, 2019).

## 2 MATERIALS AND METHODS

### 2.1 Ethical compliance

The study was approved by the Ethics Committee of the Hospital District of Southwest Finland and was conducted in accordance with the Declaration of Helsinki. All patients, aside from one who died before this study, gave their written informed consent.

### 2.2 Study population

The aim of this study was to retrospectively describe a natural course of Fabry disease caused by the R227* mutation and prospectively describe the effect of enzyme replacement therapy (ERT) in both genders with up to five years of follow‐up. This first part of the study describes the natural course of the disease, meaning the time before ERT was initiated. A group of patients who had genetic confirmation of the R227* mutation in GLA gene (the GenBank reference sequence NM_000169.2) identified in the Vaasa Hospital District or in the Hospital District of Southwest Finland during the years 2013–2014. The study population consisted of two extended families who had a common ancestor in the 19th century.

The data were collected from medical records and from the Fabry Registry (NCT00196742; sponsor: Sanofi Genzyme). Normally distributed parameters are presented as the means and standard deviations (SDs) and minimal and maximal values. Nonnormally distributed values are presented as medians and interquartile ranges (IQR 25th–75th percentiles). Categorical variables are summarized with counts and percentages. In addition, the Spearman correlation coefficient was calculated when appropriate.

Clinical examination was performed by an internist, neurologist, cardiologist, ophthalmologist, and otologist. A nephrologist was also consulted when needed. Routine laboratory tests, an electrocardiogram (ECG), a 24‐hr continuous ECG registration (Holter), imaging studies, and spiroergometry were performed at the Vaasa Central Hospital or Turku University Hospital in Finland. Interpretations of ECGs and details of laboratory parameters are presented in Figure S1. The amount of white matter lesions and the existence of ischemic strokes were assessed by a neuroradiologist (R.A.). Deep white matter lesions were classified from head MRI or CT according to the Fazekas scale (Fazekas, Chawluk, Alavi, Hurtig, & Zimmerman, 1987). Two‐dimensional transthoracic ultrasound (TTE) was performed according to the European guidelines (Evangelista et al., 2008). However, we decided to use a maximal wall thickness >12 mm instead of ≥11 mm when classifying patients' heart being hypertrophic because >12 mm is recommended in the last European consensus document for ERT in patients with Fabry disease (Biegstraaten et al., 2015). Cardiac MRI (cMRI) was performed with intravenous gadolinium infusion and analyzed by an experienced specialist in this field (M.S.; Hudsmith, Petersen, Francis, Robson, & Neubauer, 2005). Details of the imaging studies and spiroergometry are presented in Figure S2. A hearing threshold ≤ 20 dB was considered normal. Renal and cardiac biopsies were not performed.

### 2.3 The mainz severity score index

The Mainz Severity Score Index (MSSI) was used to measure the whole burden of Fabry disease. It is a validated scoring system for Fabry disease. It consists of general, neurological, cardiovascular, and renal categories. Symptoms and findings in every category result in a maximal score of 76. Fabry disease is classified as mild if the total score is less than 20. The disease burden is considered moderate if the total score is 20–40 and severe if the total score exceeds 40 (Whybra et al., 2004).

## 3 RESULTS

### 3.1 Patients

In total, 15 patients had the R227* mutation in the Vaasa and Turku districts in Finland. Four males and ten females from two different families were willing to participate in the follow‐up. An extensive family tracing revealed a probable common ancestor in the 19th century (Figures S3 and S4). The mean age at diagnosis was 46 years (*SD* 18.4; range 15–80 years). Four patients (29%) had hypertension, three patients (21%) had atrial fibrillation (AF), and two AF patients (14% of patients) had a pacemaker. None had diabetes or severe kidney disease, but one had proteinuria. Coronary angiography or coronary CT was performed for five patients because of abnormal cardiac troponin values or angina pectoris. None of the patients had significant coronary artery disease nor thinning of the epicardial vessels.

Altogether, five patients had experienced 10 significant clinical events during the natural history period (Table 1).

**Table 1:** Significant clinical events during the natural history period

ID	Gender	Age at first event	Arrhythmia	Pacemaker and indication	Stroke and/or TIA	Death
4	M	38	No	No	Stroke	No
8	F	30	Paroxysmal atrial tachycardia	No	No	No
12	F	58	AF	Slow AF	no	No
13	F	59	AF	SSS and slow AF	Silent stroke and TIA	No
14	F	73	AF	No	Stroke	Yes

Abbreviations: AF, atrial fibrillation; F, female; M, male; SSS, sick sinus syndrome; TIA, transient ischemic attack.

### 3.2 Symptoms

Fabry‐related signs and symptoms and MSSI scores are presented in Table 2. The median MSSI score in our group was 18 (IQR 10–24). The youngest male, a 15‐year‐old boy, had the lowest MSSI score. However, he had several other possible Fabry disease‐related manifestations (impaired physical growth and delayed puberty) not included in this scoring system (Hopkin et al., 2008). In addition, he had mild normocytic anemia, leucopenia, neutropenia, and intermittent thrombocytopenia in 2014. Repeated bone marrow analyses did not reveal any specific disease. Notably, the bone marrow accumulation of Gb3 had not been examined, and the contribution of Fabry disease could not be ruled out (Oliveira et al., 2008).

**Table 2:** Symptoms in the last follow‐up before initiation of enzyme replacement therapy

ID	Gender	Age at diagnosis	Neuropathic pain	Abdominal pain	Diarrhea	Clustered angiokeratoma	Sweating	Cornea verticillata	Threshold of hearing	Tinnitus	MSSI
1	M	15	Weekly	Monthly	Monthly	Yes	absent	no	<5	0	10
2	M	33	Weekly	Weekly	No	Yes	absent	N.A.	N.A.	N.A.	26
3	M	35	Daily	Weekly	Weekly	No	absent	yes	30	yes	18
4	M	36	No	Monthly	No	No	normal	yes	N.A.	N.A.	24
5	F	25	Weekly	Weekly	Weekly	No	reduced	N.A.	N.A.	N.A.	19
6	F	31	Weekly	Monthly	Monthly	No	normal	yes	15	no	5
7	F	46	no	Weekly	Weekly	No	reduced	no	25	no	10
8	F	48	No	No	No	No	normal	no	10	no	3
9	F	52	Daily	Weekly	Weekly	No	absent	yes	15	no	21
10	F	61	No	No	No	No	normal	N.A.	40	yes	10
11	F	60	Weekly	No	No	No	normal	no	20	yes	6
12	F	61	daily	No	No	No	reduced	yes	35	yes	30
13	F	66	no	No	<Monthly	No	absent	no	50	no	32
14	F	80	no	No	No	No	reduced	N.A.	N.A.	N.A.	N.A.

Abbreviations: F, female; M, male; MSSI, Mainz Severity Score Index; N.A, not available.

None had sudden hearing loss, but five out of 11 patients whose audiogram was studied before ERT had impaired hearing. Four patients suffered from tinnitus. Cornea verticillata was observed in five out of ten patients (50%) who underwent ophthalmologic examination before ERT initiation.

### 3.3 Brain imaging

**Figure 1:**
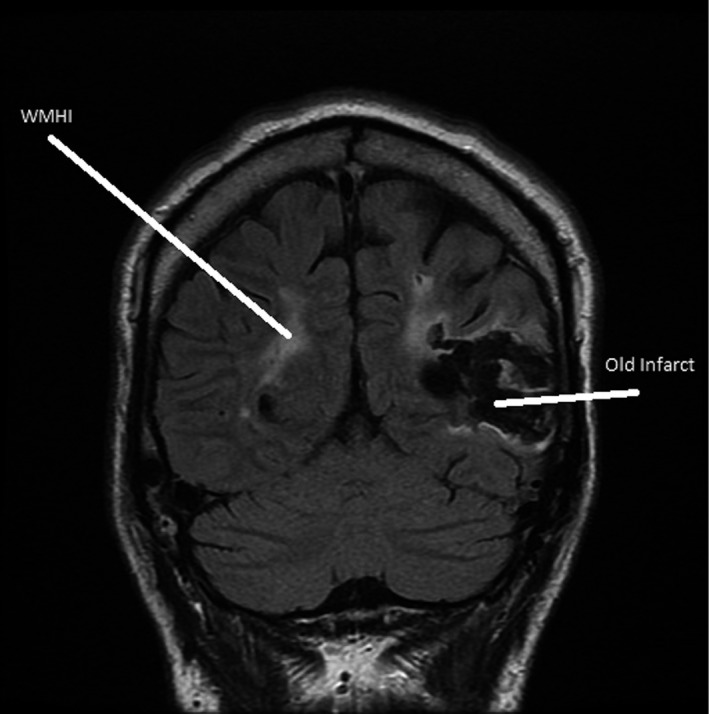
Patient with a chronic temporal lobe infarction on the left and periventricular confluent white matter hyperintensity (WMHI) on both hemispheres of the brain. These findings are typical for a patient with advanced Fabry disease

Head CT was performed on two patients and head MRI was performed on 12 patients. A 38‐year‐old male, the index patient of our cohort, experienced an acute ischemic stroke before the diagnosis of Fabry disease (Saarinen, Sillanpaa, & Kantola, 2015). One patient died of ischemic stroke at the age of 82, and another experienced a transient ischemic attack (TIA) at the age of 59 and a silent ischemic stroke sometime between ages of 59 to 69 (Table 1). All the three above‐mentioned patients had at least moderate deep white matter lesions (Fazekas ≥2). Further, one female aged 52 years showed moderate (Fazekas 2) white matter hyperintensities but no infarctions on head MRI. Seven out of 14 (50%) patients had normal head imagining without even mild (Fazekas 1) chronic small‐vessel ischemic lesions. An example of head MRI is presented in Figure 1.

### 3.4 Cardiac results

**Figure 2:**
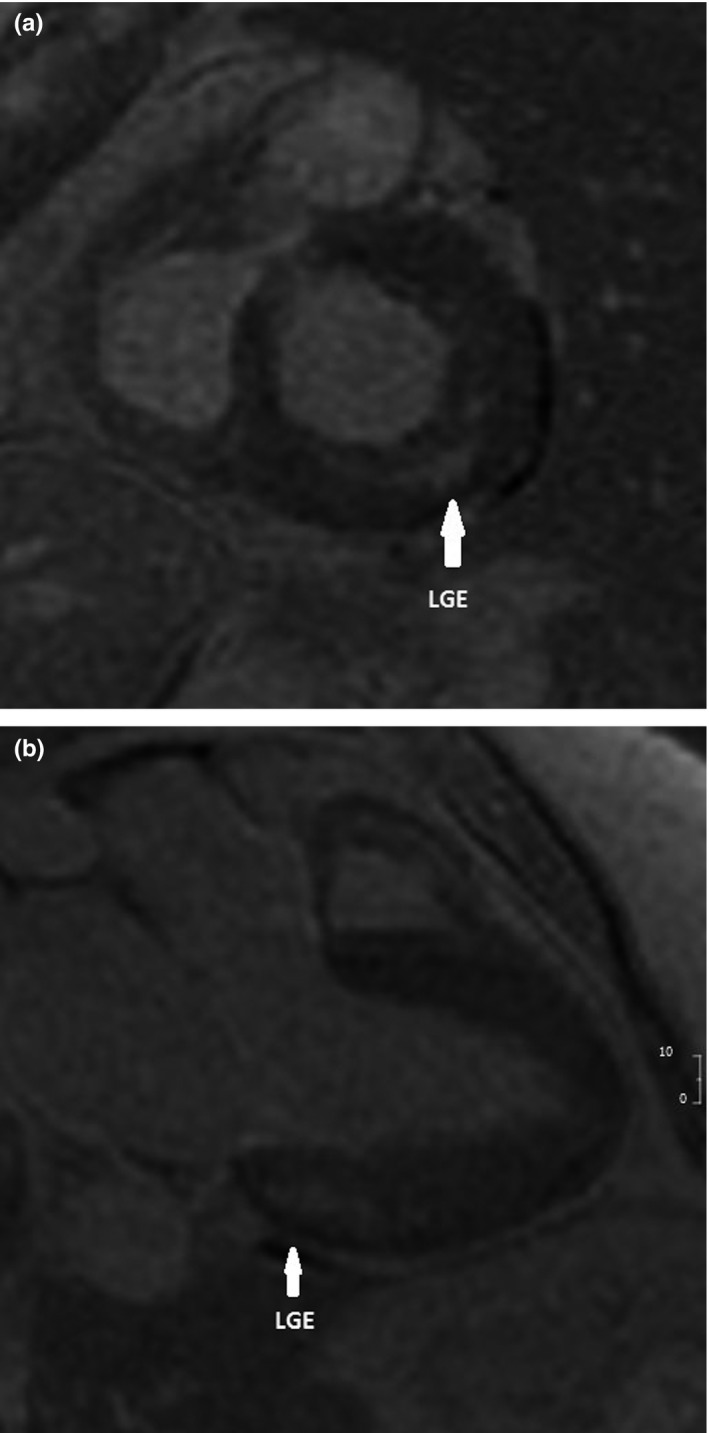
Short‐axis (a) and long‐axis (b) delayed enhanced images showing mid‐wall enhancement (white arrows) in the inferolateral wall of the left ventricle

Six patients out of 14 (43%) had a maximal wall thickness >12 mm in cMRI or in TTE, which was used in 5 patients for whom cMRI was not performed. Five out of nine patients (55%) with cMRI had late gadolinium enhancement (LGE) that varied from the size of one to two segments. Only one of them had LVMI above the reference limit. The LGE location was basal inferolateral in all patients. One patient had an additional LGE in the apical segment. Three out of five patients with LGE had cardiac troponin T (TnT) levels above the reference value. All patients without LGE had normal TnT values. One patient with LGE corresponding to the area of 1.5 segments had both a wall thicknesses <13 mm in cMRI and a TnT level <15 ng/L (Table 3). An example of cMRI with LGE is presented in Figure 2.

**Table 3:** Cardiac findings at the last follow‐up before the initiation of enzyme replacement therapy

ID	Gender	Age	Maximal wall thickness mm	LVM/BSA (g/m2)	LGE, area	TnT (ng/L)
1	M	15	6^a^	51	0	0
2	M	33	14^a^	68	0	7
3	M	35	15^b^	N.A.	N.A.	5
4	M	38	13^a^	82	1	23
5	F	25	6^a^	51	0	0
6	F	32	8^a^	59	0	6
7	F	47	9^a^	68	1.5	12
8	F	49	13^a^	62	1	7
9	F	53	9^b^	N.A.	N.A.	6
10	F	60	12^a^	87	2	92
11	F	62	9^a^	57	2	24
12	F	61	16^b^	N.A.	N.A.	100
13	F	68	21^b^	N.A.	N.A.	13
14	F	82	11^b^	N.A.	N.A.	48

Abbreviations: BSA, body surface area; F, female; LGE, late gadolinium enhancement in the area referred to size of one segment in 17 segment model; LVM, left ventricular mass in cardiac MRI; M, male; N.A., not available; TnT, cardiac troponin T.

a From cardiac MRI.

b From transthoracic ultrasound.

Electrocardiogram was normal in two out of fourteen patients. The most general findings were mild sinus bradycardia in four patients and left ventricular hypertrophy in another four patients. Two patients had AF, and one patient was on sequential pacing. Holter ECG was performed in eight patients without revealing any new arrhythmic episodes.

Spiroergometry was performed to evaluate the significance of chronotropic incompetence and airway obstruction in limiting exercise capacity. It was performed in six female patients. Two of them had mild obstruction, FEV1/FVC ratio 0.63 and 0.65, but both had normal exercise capacity. None had chronotropic incompetence.

### 3.5 Laboratory parameters

The mean estimated glomerular infiltration rate (eGFR) measured by chronic kidney disease epidemiology collaboration (CKD‐EPI) was 93.7 ml/min/1.73 m^2^ (*SD* 21.1; range 57–126 ml/min/1.73 m^2^). The urine albumin to creatinine ratio (U‐Alb/Crea) was abnormal only in two males aged 33 and 35 years. Both patients had cardiac hypertrophy but no strokes. The only female with a borderline abnormal U‐Alb/Crea ratio also had significant cardiac hypertrophy, but no cerebrovascular manifestations. Urine analysis was slightly abnormal in all seven patients for whom urine analysis results were available. Microscopic hematuria was the most common abnormal result. Concomitant IgA nephropathy and other renal diseases cannot be ruled out as no biopsies were done.

Median GLA was 71 pmol*spot/20 hr (reference 160–2000 pmol*spot/20 hr). In males, the median was 10 pmol*spot/20 hr and females 98 pmol*spot/20 hr. Median LysoGb3 in plasma was 9.7 ng/L (reference <1.8 ng/L)) and in urine was 47 µmol/mol Crea. In males and females, the values were 105 ng/L and 6 ng/L for LysoGb3 in plasma and 76 µmol/mol Crea and 24 µmol/mol Crea for LysoGb3 in urine, respectively. Higher GLA values were associated with lower plasma LysoGb3 and urine Gb3 values (Spearman correlation coefficient −0.84, *p* = .002 and −0.85, *p* = .007, respectively). Plasma LysoGb3 and urine Gb3 were positively correlated (Spearman correlation coefficient 0.709, *p* = .02; Table 4).

**Table 4:** Fabry specific laboratory parameters before the initiation of enzyme replacement therapy

ID	Gender	Age	GLA pmol* spot /20 hr	U‐Gb3 µmol/ mol Crea	LysoGb3 ng/L
1	M	15	10	52	110
2	M	33	10	288	105
3	M	35	25	69	88
4	M	38	0	83	N.A.
5	F	25	135	17	5
6	F	32	N.A.	75	6
7	F	47	115	5	4
8	F	49	153	12	N.A.
9	F	53	62	116	17
10	F	60	N.A.	20	6
11	F	62	81	19	5
12	F	61	N.A.	N.A.	N.A.
13	F	68	82	41	13
14	F	82	N.A.	N.A.	N.A.

Note

GLA, alfa galactosidase A (the GenBank reference sequence NM_000169.2); the reference range was 160–2000 pmol*spot /20 hr. U‐Gb3, globotriaosylceramide in urine; the reference value was 10 µmol/mol creatinine or less. LysoGb3, globotriaosylshingosine in plasma; the reference range was 1.8 ng/ml or less.

Abbreviations: F, female; M, male; N.A., not available.

## 4 DISCUSSION

In this study, we evaluated the natural course of Fabry disease with the confirmed nonsense mutation R227* (p.Arg227Ter, c.680C>T) in patients living in western Finland. All patients, four males and ten females, belonged to the same extended family with a common ancestor in the 19th century, which is the strength of our study because it minimized confounding factors, including environmental and cultural factors. R227* seems to be a highly pathogenic mutation in both genders. All four males had a classic Fabry disease. The natural course of the R227* mutation in females was more variable and contradictory.

In previous studies, the left ventricular mass index (LVMI) had a positive correlation with age (Arends et al., 2017; Kampmann et al., 2008). Papillary muscle hypertrophy is partly responsible for LVMI in both genders (Kozor et al., 2015, 2017). In our study, LVMI was within the range in all patients except for one. Notably, the older patients in our study were those for whom information was missing.

A continuous rise in troponin levels is known to be associated with LGE (Feustel et al., 2014; Seydelmann et al., 2016), which is in accordance with our observations. In our study, LGE was present in two females without increased troponin. This phenomenon has previously been reported by Niemann et al. (2011). A normal troponin level is insufficient to exclude early cardiac manifestation in females.

The severity of Fabry disease in females varied from classic multiorgan disease to a condition that mimics the attenuated nonclassical variant. Significant cardiac hypertrophy was detected by transthoracic ultrasound in two sisters, patients 10 and 12, in their 60s. The older sister, patient 12, had severe cardiac hypertrophy since 1993. She developed AF with a slow heart rate and received a pacemaker in 2010. She had heart failure with preserved ejection fraction and New York Heart Association (NYHA) classification class II symptoms even though she had been on sequential pacing since her last cardioversion in 2013. She also had hypohidrosis and the typical cornea verticillata. In contrast, her younger sister, patient 10, did not have any typical symptoms of classic Fabry disease and had a normal exercise performance in spiroergometry. The only subjective symptoms were mild tinnitus and hearing impairment with a hearing threshold of 40 dB. On clinical examination, a few angiokeratomas were found. However, the presence of some nonclustered angiokeratomas alone is insufficient for classifying classical Fabry disease (van der Tol et al., 2014). She could not be considered an asymptomatic mutation carrier because of the significant cardiac hypertrophy that was confirmed by cMRI.

The attenuated cardiac variant of Fabry disease has previously been described in males in Japan and in the United States (Nakao et al., 1995; von Scheidt et al., 1991). One cardiac variant of Fabry derives from the N215S mutation. Oder et al. examined 26 patients, 13 males, and 13 females with a mean age of 49 years, who were originally misdiagnosed for ordinary HCM and generally possessed sarcomeric mutations. Cardiac hypertrophy was mild even if LGE was present in 18 out of the 26 patients (69%). Alfa‐galactosidase was mildly reduced and lysoGb3 was slightly above the upper normal limit in 19 out of the 26 patients (Oder et al., 2017). However, it is possible that other organ manifestations develop later in the life span (Lavalle et al., 2018). Recently, Arends et al. (2017) and Pan et al. (2016) noted that gender and genotype are essential but not unique factors that affect the phenotype. Studies of attenuated variants of Fabry disease suggest that patients 6, 8, and 14 in our group had Fabry disease severity similar to attenuated cardiac variants. Females with an attenuated nonclassical disease course have a better prognosis than classical females but a worse prognosis than healthy mutation carriers (Arends et al., 2017). That is important for motivating asymptomatic patients to follow‐up regularly and determining the right time to begin ERT when there is evidence of early damage in critical organs, such as the heart, brain, or kidneys.

One limitation of our study is the homogenous study population. The results of our study probably cannot be generalized to the patients with the same R227* mutation but with a different background. It is possible that the absence of significant renal disease is accidental and depends on the relatively young age of males in our study or some other genetic or environmental renoprotective factors. However, genotype–phenotype correlation studies are often hampered by confounding factors that are difficult to handle by statistical methods, especially in rare diseases with several mutations. In fact, it might be one reason for insufficient information in this field.

The second limitation is the lack of information on the X‐chromosome inactivation pattern that can at least partially explain the attenuated nonclassical disease course in females.

The third limitation is the incomplete data of some patients. In some patients, ERT was initiated before the LysoGb3 measurement was available. Two females with older non‐MRI compatible pacemakers lacked all MRI data, and two males with severe symptoms began ERT before cMRI was performed. The patient who died before the onset of this study refused cMRI. Thus, it is probable that the percentage of patients with LGE is underestimated in our study.

In conclusion, our study confirmed classic Fabry disease in males with the R227* mutation. The disease burden in females did not vary from asymptomatic mutation carrier to severe disease but from attenuated cardiac variant‐like disease to severe classic disease. Females can be easily misclassified as asymptomatic mutation carriers and withdraw from regular follow‐up and therapy.

A natural progression of our study would be to combine our data with other R227* mutation data from different populations with different genetic backgrounds. If the results are generalizable for this mutation, further research should also be undertaken to investigate whether the attenuated disease course is also present with other mutations causing classic Fabry disease. In addition, the role of X‐chromosome inactivation in Fabry disease severity is still unanswered and should be further investigated.

## CONFLICT OF INTEREST

Dr. Pietilä‐Effati served on the advisory committees for Sanofi‐Genzyme and Shire, participated in a clinical study sponsored by Sanofi‐Genzyme, received research support from Sanofi‐Genzyme, received speaker fees from Sanofi‐Genzyme, and received travel support from Sanofi‐Genzyme and Shire. Dr. Saarinen has received speaker honoraria from Sanofi‐Genzyme and Shire, funding for travel from Sanofi‐Genzyme and Shire, and research support from Sanofi‐Genzyme and has participated in the scientific advisory board of Sanofi‐Genzyme. M. Sc. Löyttyniemi: no conflicts of interest. Dr. Autio: no conflicts of interest. Dr. Saarenhovi: no conflicts of interest. Dr. Haanpää: no conflicts of interest. Dr. Kantola has received speaker honoraria from Sanofi‐Genzyme and Shire; funding for travel from Amicus, Sanofi‐Genzyme and Shire; research support from Sanofi‐Genzyme and Shire; and has participated in the scientific advisory board of Amicus and Sanofi‐Genzyme.

## AUTHORS' CONTRIBUTIONS

Päivi Pietilä‐Effati was involved in the study design, data collection, data analysis, and manuscript drafting. Jukka T. Saarinen was involved in the study design, data interpretation and drafting, and revising the manuscript for intellectual content. Eliisa Löyttyniemi was involved in the statistical analysis and revising the manuscript for intellectual content. Reijo Autio was involved in the analysis and interpretation of the data and revising the manuscript for intellectual content. Maria Saarenhovi was involved in data analysis and revising the manuscript for intellectual content. Maria K. Haanpää was involved in data analysis and revising the manuscript for intellectual content. Ilkka Kantola was involved in data analysis and drafting and revising the manuscript. All authors have read, edited, and approved the final manuscript.

## Data Availability

The data that support the findings of this study are available on request from the corresponding author. The data are not publicly available due to privacy or ethical restrictions.
