# Supplementary figures and images for: Natural course of Fabry disease with the p. Arg227Ter (p.R227*) mutation in Finland: Fast study
Source: Mol Genet Genomic Med. 2019 Aug 14;7(10):e00930. doi: 10.1002/mgg3.930 (PMC6785458; doi:10.1002/mgg3.930)

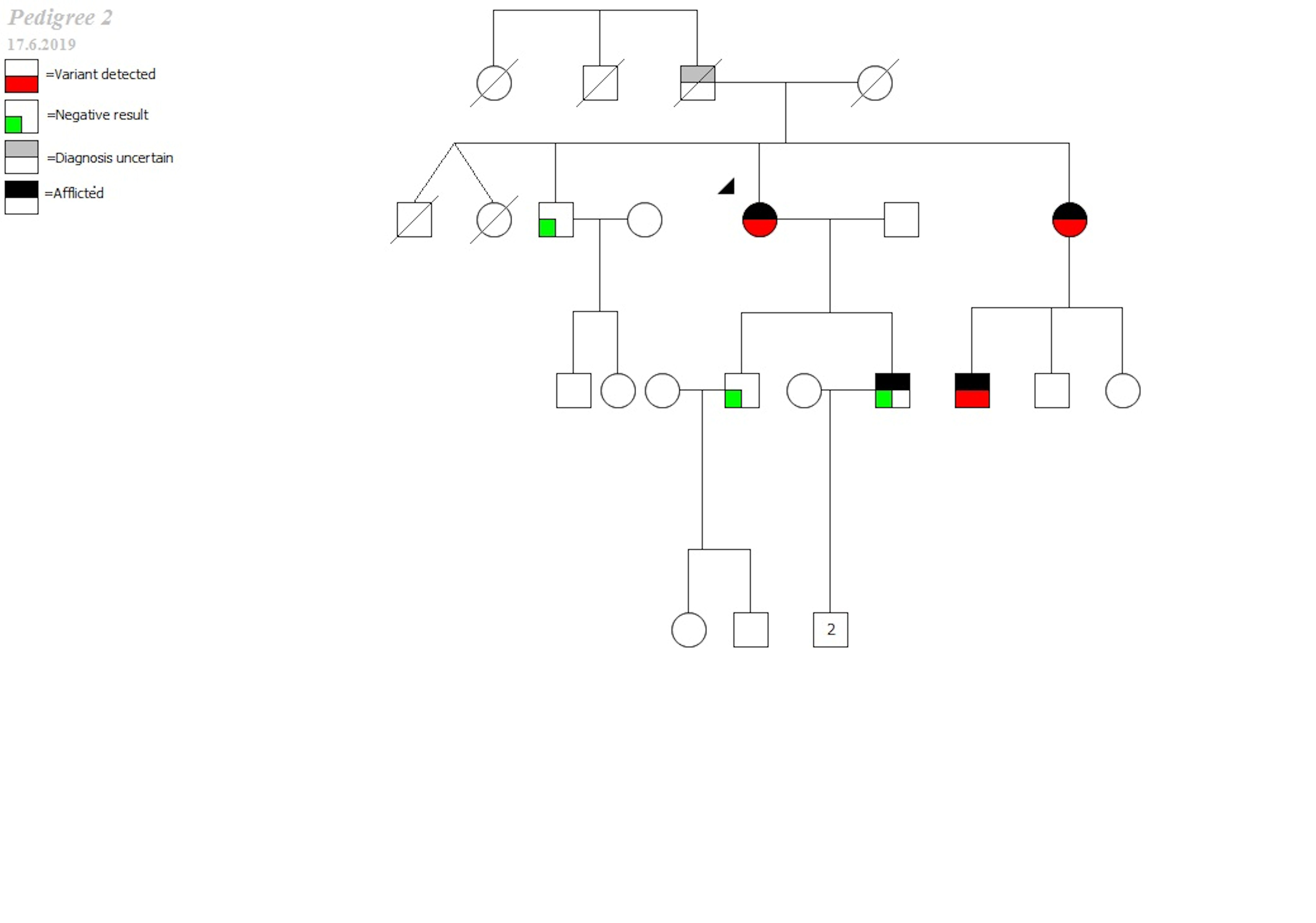

Supplement: Supplementary file 2 [file MGG3-7-e00930-s002.jpg]
