# Supplementary material for: Natural course of Fabry disease with the p. Arg227Ter (p.R227*) mutation in Finland: Fast study
Source: Mol Genet Genomic Med. 2019 Aug 14;7(10):e00930. doi: 10.1002/mgg3.930 (PMC6785458; doi:10.1002/mgg3.930)
Supplement: Supplementary file 3 [file MGG3-7-e00930-s003.docx]

**Supplementary material 1**

**ECG**

Sinusbradycardia is defined as heart rate <60 beats per minute at rest. LVH is defined according to Cornell and Sokolow-Lyon criteria. Wide QRS is defined as QRS duration >100 ms. Short PR-interval is defined as PR interval <120 ms.

**Laboratory values**

Reference for troponin T (TnT) was <15 ng/l.

N-terminal pro-brain-type natriuretic peptide (NT-Pro-BNP) references were age and sex dependent. In our cohort reference were <84 ng/l for males less than 50 years old. For females references are <155, <222, <285 and <738 ng/l in ages less than 50, 50-64, 65-75 and over 75 years old, respectively.

Reference value for creatinine is 60-100 µmol/l. Reference values for eGFR measured by CDK-EPI formula are: >90 normal, 60-89 slightly decreased, 30-59 moderately decreased, 15-29 severely decreased, < 15 ml/min/1.73m² end stage.

Reference value for cystatin C is 0.00-1.20 mg/l at the age of 16-64 years and 0.7-1.53 mg/l at the age of 65-74 years.

Reference value for U-Alb/Crea is <2.5 mg/mmol for males and <3.5 mg/mmol for females.

Alfa galactosidase A (GLA) was measured from a dried blood spot in Hamburg University Medical Center.

LysoGb3 was measured from a dried blood spot in Centogene AG (Rostock, Germany).

U-Gb3 was measured in Sahlgrenska University Hospital (Göteborg, Sweden).
