# Supplementary material for: Natural course of Fabry disease with the p. Arg227Ter (p.R227*) mutation in Finland: Fast study
Source: Mol Genet Genomic Med. 2019 Aug 14;7(10):e00930. doi: 10.1002/mgg3.930 (PMC6785458; doi:10.1002/mgg3.930)
Supplement: Supplementary file 4 [file MGG3-7-e00930-s004.docx]

**Supplementary material 2**

**Cardiac MRI**

Cardiac MRI was performed using a 1.5 Tesla full-body MRI scanner (GE Discovery MR450; Boston, United States of America). Left ventricular (LV) volume, wall thickness and ejection fraction were determined from Fiesta-cine imagining. Papillary muscles were included in the LV mass. The mean normal values for LV mass indexed by body surface area (BSA) (g/m2) were 67 ± 10 for males < 35 years old and 60 ± 9 for males ≥ 35 years old. For females the mean normal values were 53 ± 9 and 52 ± 9, respectively. Upper reference values were calculated mean + 2 SD.

Late gadolinium enhancement (LGE) images were acquired 12-17 min after intravenous injection (Dotarem® 279,3mg/ml) that was given 20-30 ml depending on patient`s weight. The amount of LGE was presented as an area corresponding segment size, for example “LGE was found in the area corresponding the size of two out of seventeen segments”. The location of LGE was given using the same seventeen segment model where basal parts of left ventricle were numbered from 1 to 6, medial parts from 7-12, distal parts from 13-16 and the most apical part was number 17^1,2^.

**Head MRI**

Head MRI was performed using a 1.5 Tesla full-body MRI scanner (GE Discovery MR450; Boston, United States of America or GE Optima MR450W; Boston, Unites States of America). Presence and amount of white matter lesions and ischemic lesions were defined ^3^. All of the patients were imaged at least following sequences: T2 fast spin echo, FLAIR, T1 spin echo, SWAN (gradient echo hemo sequence) and DWI (diffusion weighted imaging). Some of the patients were also imaged with T1 gadolinium (15 ml gadoterate meglumine i.v.) sequence and 3D time of flight (TOF). This variation in protocols was due to differing clinical symptoms and suspected etiology.

**Cardiac transthoracic ultrasound**

Cardiac transthoracic ultrasound was performed (Vivid 7 [3.5 MHz], GE Vingmed Ultrasound AS, Horten, Norway) patient laying on the left side. Wall thickness in septum (IVS) and in posterior wall (PW) and end-diastolic and end-systolic dimensions (LVEDD and LVESD, respectively) were measured from parasternal long axis or short axis using M-mode in the level of the tips of the mitral valve leaflet. LV ejection fraction (EF) was calculated from M-mode using Teichholz formula. Diastolic function was measured with pulsed Doppler using the ratio of early-to-late (E/A) diastolic flow velocity and the deceleration time. These measurements were taken from apical four chamber view from the level of the tips of the mitral valve leaflets. The left ventricular filling pressure was determined with the ration E/E´ where E´ represents early diastolic movement of mitral annulus. E´ was calculated as a mean value of medial E´ and lateral E´ if the both values were reliable (angulation < 15 degrees)^4^.

**Spiroergometry**

Cardiopulmonary exercise test was performed by the Oxycon Pro® (Jäger, Würzburg, Germany). It was used to measure maximal pulmonary oxygen consumption (VO_2_ ml/kg/min) and its relation to maximal oxygen consumption (VE/VO2). The measured values were related to age. Forced expiratory volume in one second (FEV_1_) and its relation to forced vital capacity (FVC), maximal expiratory flow in middle size (MEF50) and small size airways (MEF25) were also determined^5^. FEV_1_/FVC ratio less than 0.70, was considered obstructive according to Global Initiative for Chronic Obstructive Lung Disease (GOLD) guidelines ^6^.

1. Hudsmith LE, Petersen SE, Francis JM, Robson MD, Neubauer S. Normal human left and right ventricular and left atrial dimensions using steady state free precession magnetic resonance imaging. *J Cardiovasc Magn Reson*. 2005;7(5):775-782.

2. Kawel N, Turkbey EB, Carr JJ, et al. Normal left ventricular myocardial thickness for middle-aged and older subjects with steady-state free precession cardiac magnetic resonance: The multi-ethnic study of atherosclerosis. *Circ Cardiovasc Imaging*. 2012;5(4):500-508. Accessed Jan 13, 2019. doi: 10.1161/CIRCIMAGING.112.973560.

3. Fazekas F, Chawluk JB, Alavi A, Hurtig HI, Zimmerman RA. MR signal abnormalities at 1.5 T in alzheimer's dementia and normal aging. *AJR Am J Roentgenol*. 1987;149(2):351-356.

4. Evangelista A, Flachskampf F, Lancellotti P, et al. European association of echocardiography recommendations for standardization of performance, digital storage and reporting of echocardiographic studies. *Eur J Echocardiogr*. 2008;9(4):438-448.

5. Rietjens GJ, Kuipers H, Kester AD, Keizer HA. Validation of a computerized metabolic measurement system (oxycon-pro) during low and high intensity exercise. *Int J Sports Med*. 2001;22(4):291-294. Accessed Nov 13, 2018. doi: 10.1055/s-2001-14342.

6. Rabe KF, Hurd S, Anzueto A, et al. Global strategy for the diagnosis, management, and prevention of chronic obstructive pulmonary disease: GOLD executive summary. *Am J Respir Crit Care Med*. 2007;176(6):532-555.
